# Platelet Activation and Aggregation Induced by Streptococcus bovis*/*Streptococcus equinus Complex

**DOI:** 10.1128/spectrum.01861-22

**Published:** 2022-11-14

**Authors:** Gustav Pernow, Oonagh Shannon, Jonas Öberg, Bo Nilson, Magnus Rasmussen

**Affiliations:** a Division of Infection Medicine, Department of Clinical Sciences Lund, Lund University, Helsingborg Hospital, Lund, Sweden; b Division of Infection Medicine, Department of Clinical Sciences Lund, Lund University, Lund, Sweden; c Department of Clinical Microbiology, Infection Control and Prevention, Office for Medical Services, Region Skåne, Lund, Sweden; d Division of Medical Microbiology, Department of Laboratory Medicine Lund, Medical Faculty, Lund University, Office for Medical Services, Region Skåne, Lund, Sweden; e Division of Infection Medicine, Department of Clinical Sciences Lund, Lund University, Skåne University Hospital, Lund, Sweden; Ohio State University

**Keywords:** *Streptococcus bovis/Streptococcus equinus* complex, infective endocarditis, platelet activation, bacteria-platelet interactions, subspecies, IgG-Fc-receptor

## Abstract

Streptococcus bovis*/*Streptococcus equinus complex (SBSEC) is a common cause of infective endocarditis (IE). For IE-pathogens, the capacity to activate and aggregate platelets is believed to be an important virulence mechanism. While the interactions between bacteria and platelets have been described in detail for many Gram-positive pathogens, little research has been carried out with SBSEC in this respect. Twenty-six isolates of the four most common species and subspecies of SBSEC identified in bacteremia were collected, and interactions with platelets were investigated in platelet rich plasma (PRP) from three donors. Aggregation was studied using light-transmission aggregometry and platelet activation using flow cytometry detecting surface upregulation of CD62P. Platelets and serum were treated with different inhibitors to determine mechanisms involved in platelet aggregation and activation. Twenty-two of 26 isolates induced aggregation in at least one donor, and four isolates induced aggregation in all three donors. In PRP from donor 1, isolate SL1 induced a rapid aggregation with a median time of 70 s to reach 50% aggregation. Blockade of the platelet Fc-receptor or enzymatic cleavage of IgG abolished platelet activation and aggregation. The capacity for bacteria-induced platelet aggregation was also shown to be transferable between donors through serum. SBSEC mediates platelet aggregation in an IgG and IgG-Fc-receptor dependent manner. Bacterial activation of platelets through this pathway is common for many bacteria causing IE and could be a potential therapeutic target for the prevention and treatment of this infection.

**IMPORTANCE** The capacity of bacteria to activate and aggregate platelets is believed to contribute to the pathogenesis of IE. The Streptococcus bovis/Streptococcus equinus complex (SBSEC) contains known IE-pathogens, but there is limited research on the different subspecies ability to interact with platelets and what signaling pathways are involved. This study reports that 22 of 26 tested isolates of different subspecies within SBSEC can induce aggregation, and that aggregation is host dependent. The Fc-IgG-receptor pathway was shown essential for platelet activation and aggregation. To the best of our knowledge, this is the first study that reports on platelet interactions of SBSEC-isolates other than Streptococcus gallolyticus subspecies *gallolyticus* as well as the first study to report of mechanisms of platelet interaction of SBSEC-isolates. It adds SBSEC to a group of bacteria that activate and aggregate platelets via the platelet Fc-receptor. This could be a potential therapeutic target for prevention of IE.

## INTRODUCTION

The Streptococcus bovis/Streptococcus equinus complex (SBSEC) contains several species of bacteria. They belong to the nonenterococcal Lancefield group D streptococci and are commensals that can be found in the intestinal flora of healthy individuals (1). They are also opportunistic pathogens and are associated with diseases such as infective endocarditis (IE), colonic pathology, and hepatobiliary disease (2–5). Since Schlegel et al. (6) introduced a new taxonomy in 2003, SBSEC has undergone a change in nomenclature. Today the group is divided into seven species and subspecies, of which Streptococcus gallolyticus ssp. *gallolyticus* (*Sg gallolyticus)* is most often associated with IE. However, in part due to difficulties with correct identification of these subspecies, the clinical characteristics of different isolates is not fully understood (7).

IE is a severe condition associated with a risk of complications such as stroke, other types of embolization, and heart failure. In-hospital mortality remains high at around 20%, despite advances in diagnostics and treatment. SBSEC has been determined to be the causative agent for IE in 6% of cases overall and 10% in Europe (3). SBSEC IE is a more common disease among elderly patients and affects men more than women (2, 8–10). Usually, the infection occurs on native valves, but SBSEC are also reported in cases of prosthetic valve IE (2, 8–12).

To cause IE, the bacteria need to access the bloodstream and adhere to the endothelium of the valves. This adhesion can occur when bacteria bind exposed fibrin-platelet thrombi on the valves after endothelial injury or bind to inflamed endothelial cells via platelets (13, 14). It has also been hypothesized that adhesion can occur on undamaged valves through small circulating thrombi caused by bacteria (15).

In these scenarios, the bacteria–platelet interaction is of interest because platelets have a central role in thrombus formation. Platelets express surface receptors for collagen (GPV1), fibrinogen (GPIIb/IIIa), and von Willebrand factor (GPIb/IX/V) through which they can adhere to sites of endothelium damage and become activated. When activated, the GPIIb/IIIa receptor is able to bind to other platelets via a fibrinogen bridge and cause aggregation. The activated platelet also secretes granules of substances that activate nearby platelets (16). Several IE pathogens have been investigated in respect to platelet interactions. For S. aureus, the capacity for platelet activation and aggregation is dependent upon host factors such as IgG and the platelet Fc-receptor, and bacterial surface proteins such as clumping factor (17, 18). Different streptococci have also been demonstrated to interact with platelets. For Streptococcus sanguinis, one of the more common viridans group streptococci in IE, several mechanisms for platelet interactions have been described. Some isolates, expressing the surface protein SrpA, are able to bind directly to the GPIb-receptor on platelets and provoke rapid aggregation (19, 20). In the absence of SrpA, S. sanguinis can still trigger aggregation in an IgG- and complement-dependent manner, although not as rapid as direct binding. The capacity of S. sanguinis to aggregate platelets has also been shown to serve as a virulence mechanism in experimental IE (21). Moreover, Streptococcus pyogenes, Streptococcus pneumoniae, Enterococcus faecalis, and Aerococcus urinae have also been shown to activate and aggregate platelets in an IgG-dependent manner (22–25).

Clearly, many IE pathogens share the capacity to activate and aggregate platelets, and it is believed to be a virulence mechanism for bacteria. On the other hand, platelets are also part of the host defenses against infections and release antimicrobial peptides and inflammatory mediators when activated (26). The exact role of platelet-bacteria interactions in the pathogenesis of IE is yet to be determined.

For SBSEC, there is limited research regarding the interactions with platelets. Veloso et al. (27) showed that a strain of *Sg gallolyticus* induced platelet aggregation, and a study by Grimm et al. (28) suggested that aggregation is both host and isolate dependent. However, no studies have investigated which host and bacterial factors are important for this interaction. In this study, we investigate the capacity of SBSEC isolates to activate and aggregate platelets and what mechanisms are involved in the process.

## RESULTS

### Isolate and donor-specific aggregation by SBSEC.

Twenty-six isolates of SBSEC were tested for platelet aggregation in platelet rich plasma (PRP) from three donors. The isolates selected belong to the four most common species and subspecies of SBSEC seen in the clinic (*Sg gallolyticus* [SGG1-SGG7], *Streptococcus gallolyticus* ssp. *pasterianus* [SGP1-SGP6], *Streptococcus lutentiensis* [SL1-SL7], and *Streptococcus infantarius* ssp. *infantarius* [SII1-SII6]). Table 1 shows the species and clinical conditions caused by each isolate.

**TABLE 1:** Species and clinical diagnosis for the SBSEC blood isolates^*a*^

Isolate	Species	Clinical diagnosis	Strain ID
SGG1	S. gallolyticus ssp*. gallolyticus*	Possible IE	CMRS100037
SGG2	S. gallolyticus ssp*. gallolyticus*	Definite IE^*b*^	CMRS100048
SGG3	S. gallolyticus ssp*. gallolyticus*	Definite IE	CMRS100076
SGG4	S. gallolyticus ssp*. gallolyticus*	BUO	CMRS100128
SGG5	S. gallolyticus ssp*. gallolyticus*	Intestinal focus	CMRS100123
SGG6	S. gallolyticus ssp*. gallolyticus*	BUO	CMRS100129
SGG7	S. gallolyticus ssp*. gallolyticus*	BUO	CMRS100113
SGP1	S. gallolyticus ssp*. pasteurianus*	Intestinal focus	CMRS100038
SGP2	S. gallolyticus ssp*. pasteurianus*	Colitis	CMRS100102
SGP3	S. gallolyticus ssp*. pasteurianus*	BUO	CMRS100114
SGP4	S. gallolyticus ssp*. pasteurianus*	Cholecystitis	CMRS100116
SGP5	S. gallolyticus ssp*. pasteurianus*	BUO	CMRS100124
SGP6	S. gallolyticus ssp*. pasteurianus*	Cholecystitis, POI	CMRS100068
SL1	*S. lutetiensis*	Definite IE	CMRS100049
SL2	*S. lutetiensis*	BUO	CMRS100050
SL3	*S. lutetiensis*	Liver abscess	CMRS100110
SL4	*S. lutetiensis*	BUO	CMRS100119
SL5	*S. lutetiensis*	Intestinal ischemia	CMRS100118
SL6	*S. lutetiensis*	CVCRB	CMRS100111
SL7	*S. lutetiensis*	Colitis	CMRS100126
SII1	*S. infantarius* ssp*. infantarius*	Cholangitis	CMRS100044
SII2	*S. infantarius* ssp*. infantarius*	BUO	CMRS100131
SII3	*S. infantarius* ssp*. infantarius*	Definite IE	CMRS100134
SII4	*S. infantarius* ssp*. infantarius*	BUO	CMRS100121
SII5	*S. infantarius* ssp*. infantarius*	BUO	CMRS100108
SII6	*S. infantarius* ssp*. infantarius*	BUO	CMRS100107

a IE, infective endocarditis; BUO, bacteremia of unknown focus; CVCRB, central venous catheter related bacteremia; POI, postoperative infection.

b According to the 2015 ESC Guidelines (38).

The results from aggregometry are illustrated in Table 2. In donor 1, four isolates of 26 induced aggregation, with isolate SL1 and SII6 inducing a more rapid response. In donor 2, 12/26 isolates induced aggregation, and in donor 3, 21/26 isolates caused aggregation. Four isolates induced platelet aggregation in all three donors, as did the positive-control bacteria S. pyogenes AP1. Four isolates did not induce aggregation in any donor.

**TABLE 2:** Platelet aggregation induced by SBSEC isolates

	Donor 1		Donor 2		Donor 3	
Isolate^*b*^	Aggregation (%)	T_50_ (min:sec)	Aggregation (%)	T_50_ (min:sec)	Aggregation (%)	T_50_ (min:sec)
AP1	71%	4:48	82%	02:21	78%	03:39
SGG1	-^*a*^	-	63%	03:45	41%	06:25
SGG2	-	-	68%	08:30	-	-
SGG3	-	-	52%	07:30	54%	10:25
SGG4	-	-	-	-	45%	06:55
SGG5	-	-	57%	10:46	42%	04:59
SGG6	73%	03:18	56%	17:30	46%	07:53
SGG7	-	-	-	-	54%	05:43
SGP1	-	-	48%	05:00	48%	07:10
SGP2	-	-	-	-	54%	07:56
SGP3	-	-	-	-	-	-
SGP4	-	-	-	-	-	-
SGP5	-	-	-	-	-	-
SGP6	-	-	-	-	-	-
SL1	66%	01:26	60%	09:50	49%	05:35
SL2	-	-	55%	07:00	64%	05:00
SL3	-	-	-	-	59%	04:44
SL4	-	-	-	-	66%	05:52
SL5	-	-	-	-	82%	05:48
SL6	-	-	-	-	68%	06:22
SL7	-	-	-	-	49%	04:51
SII1	-	-	65%	07:05	76%	05:55
SII2	-	-	-	-	55%	02:57
SII3	76%	07:26	53%	04:27	45%	03:59
SII4	-	-	-	-	46%	06:55
SII5	-	-	43%	05:03	87%	05:44
SII6	81%	01:08	55%	10:05	68%	07:06

a (-) indicates less than 40% aggregation.

b *n* = 1 for all isolates in each donor.

**FIG 1:**
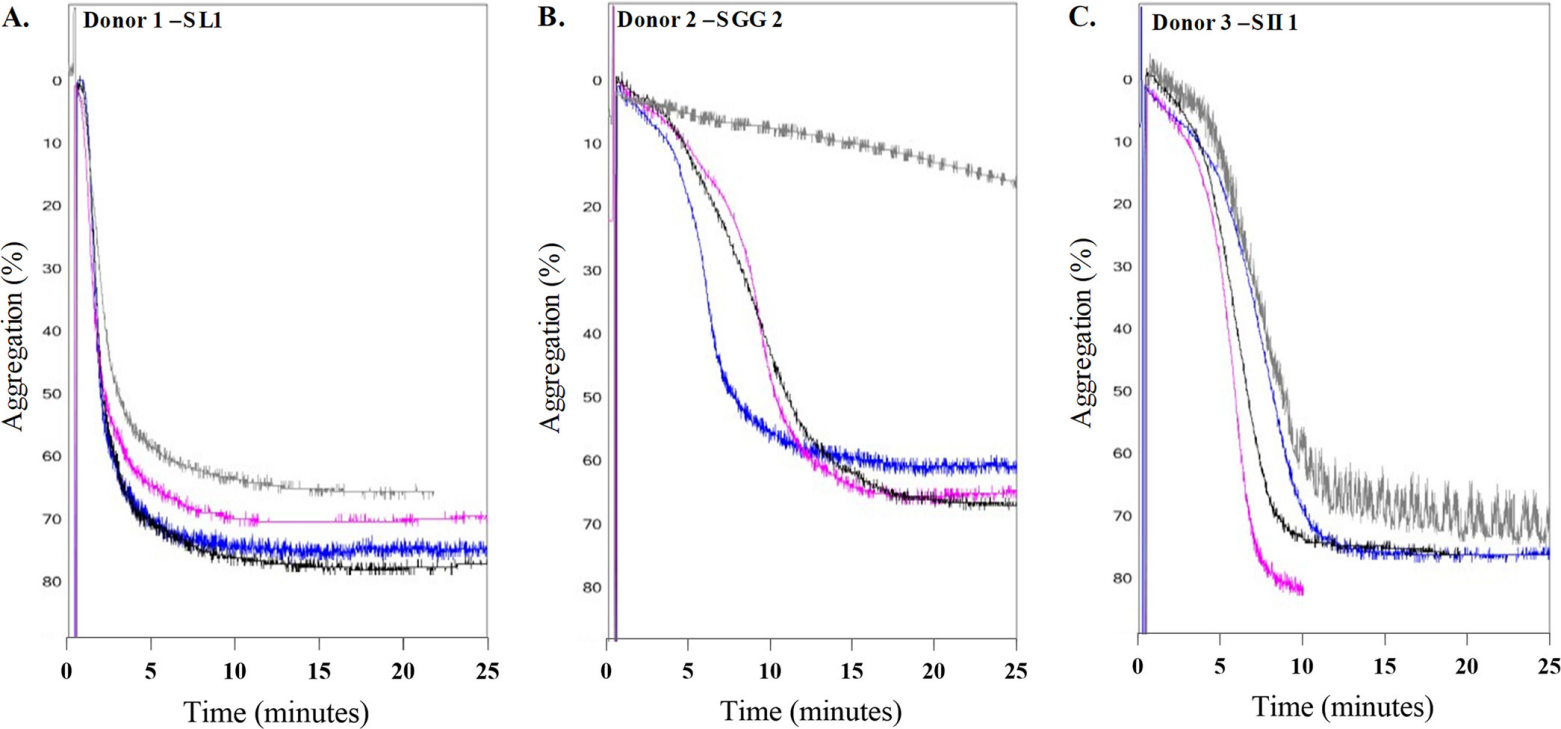
SBSEC isolates induce aggregation with different lag times. Bacteria were added to PRP and aggregation was monitored using a chrono log aggregometer. Data are presented for aggregation by the same isolate in PRP from the same donor from four independent experiments. The *y* axis represents the change in light transmission, with 0% transmission representing PRP after addition of bacteria and PPP as the baseline for 100% light transmission. As the platelets aggregate, the sample absorbs less light. Time was measured for 50% of the curve’s amplitude (T_50_). Each line shows the aggregation from one experiment. SL1 induces aggregation in donor 1 with a median T_50_ of 01:10 (min:sec) (A). SGG2 induces aggregation in donor 2 with a median T_50_ of 08:00 (B). SII1 induces aggregation in donor 3 with a median T_50_ of 05:50 (C).

Aggregation caused by SL1 in donor 1, SGG2 in donor 2, and SII1 in donor 3 were chosen for further investigation, as these isolate-donor combinations resulted in clear aggregation that was not affected by changes in bacterial concentrations during preliminary experiments. Fig. 1 shows aggregation curves of SL1 in PRP from donor 1, SGG2 in donor 2, and SII1 in donor 3 from four independent experiments. SL1 induced a very rapid aggregation in donor 1 with a median time to 50% of maximum aggregation (T_50_) of 01:10 (min:sec). SGG2 induced aggregation with a median T_50_ of 08:00 in donor 2, and SII1 induced aggregation in donor 3 with a median T_50_ of 05:50.

### Platelet aggregation by SBSEC is dependent on IgG.

**FIG 2:**
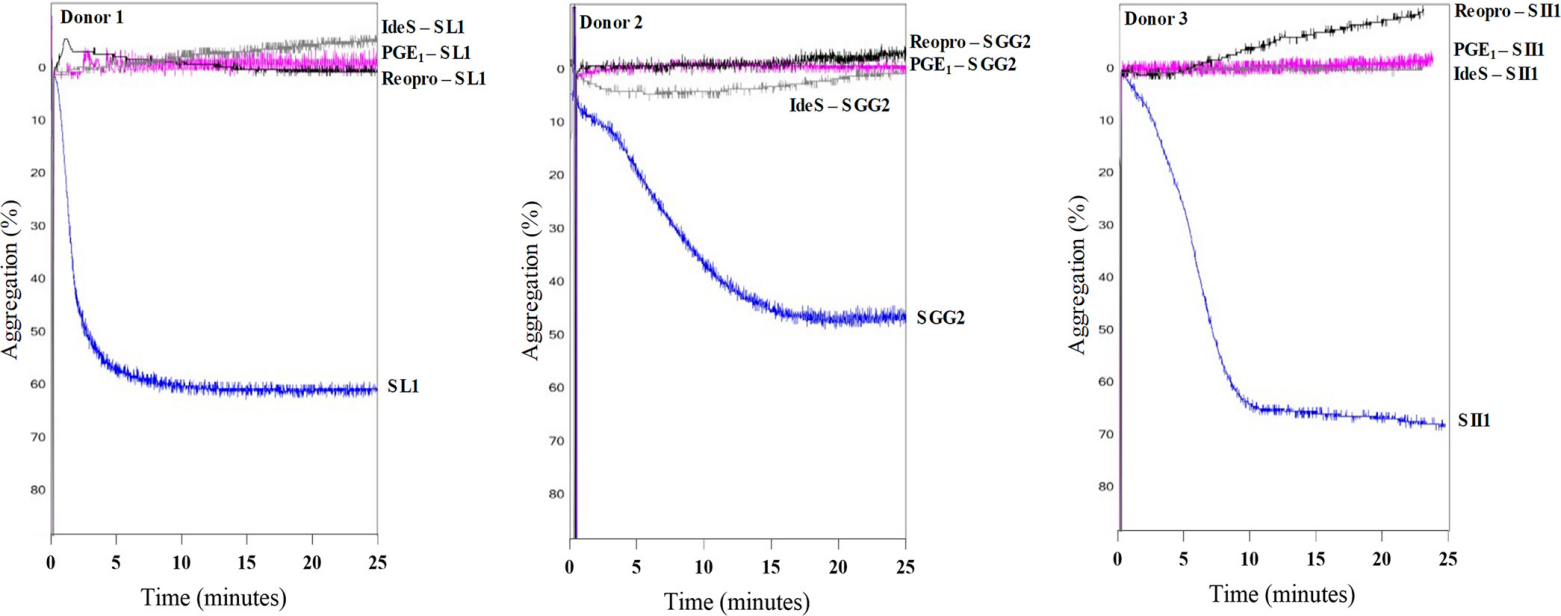
Aggregation mediated by SBSEC isolates is dependent on IgG. Bacteria were added to PRP and aggregation was monitored using a chrono log aggregometer. The *y* axis represents the change in light transmission, with 0% transmission representing PRP after addition of bacteria and PPP as the baseline for 100% light transmission. As the platelets aggregate, the sample absorbs less light. The aggregation curves of SL1 in PRP from donor 1, SGG2 in donor 2, and SII1 in donor 3 with bacteria added to either PRP alone or PRP pretreated with prostaglandin E_1_ (PGE_1_), ReoPro, or IdeS. The experiment was repeated in donor 1 and 2 with similar results.

To assess the role of plasma factors in platelet aggregation by SBSEC, PRP was treated with specific blockers. The aggregometry curves for SL1, SGG2, and SII1 can be seen in Fig. 2. Addition of the platelet inhibitor PGE_1_ to PRP blocked aggregation in all donors, showing that aggregation was not a result of passive agglutination. As expected, blocking of the platelet fibrinogen receptor, using a monoclonal antibody (Reopro), resulted in loss of aggregation in all donors. PRP was also pretreated with the specific IgG-degrading enzyme IdeS of S. pyogenes. In PRP pretreated with IdeS, all three isolates lost their ability to aggregate platelets, indicating that aggregation is IgG dependent.

### Platelet aggregation is transferable between donors by serum.

**FIG 3:**
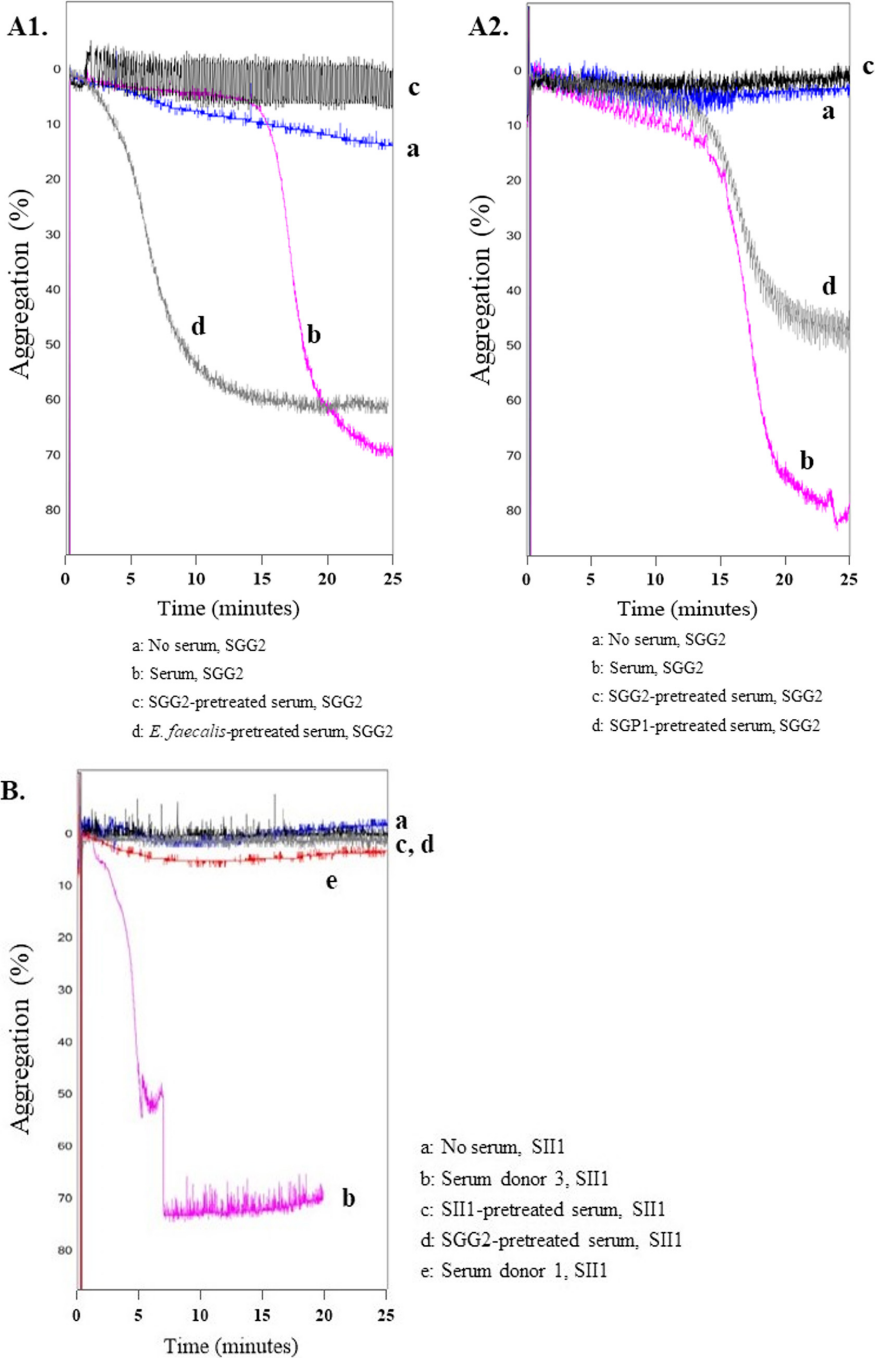
Aggregation mediated by SBSEC isolates is transferable through serum. Bacteria were added to PRP and aggregation was monitored using a chrono log aggregometer. Aggregation curves of three experiments with bacteria added to samples with either PRP alone, PRP with serum, or PRP with serum pretreated with bacteria. PRP in all samples was from donor 1. Two experiments are with isolate SGG2 and serum from donor 2 (A1, A2), and one experiment with isolate SII1 and serum from donor 3 (B). The *y* axis represents the change in light transmission, with 0% transmission representing PRP after addition of bacteria and PPP as the baseline for 100% light transmission. As the platelets aggregate, the sample absorbs less light. The figure shows the results of three separate experiments. (A) SGG2 added to PRP from donor 1 alone (A1-curve a, A2-curve a) does not induce aggregation. In samples with serum added (A1-curve b, A2-curve b) aggregation occurs, but the transferability of aggregation is taken away if the serum is pretreated with SGG2 (A1-curve c, A2-curve c). If the serum is pretreated with E. faecalis (A1-curve d) or SGP1 (A2-curve d) aggregation remains transferable through serum. (B) SII1 added to donor 1 alone (curve a) does not induce aggregation. With addition of serum, the isolate induces aggregation (curve b). If the serum is pretreated with SII1(curve c) or SGG2 (curve d), addition of SII1 does not cause aggregation. Addition of serum from donor 1 does not lead to isolate-induced aggregation (curve e).

There were some isolates that did not induce aggregation in donor 1 but did aggregate platelets in the other donors. This was an opportunity to investigate whether the ability to aggregate platelets could be transferred between donors through serum. Serum was collected from donor 2 and 3 and added to PRP from donor 1 before the addition of bacteria (Fig. 3). Once again, when the isolates (SGG2 or SII1) were added to PRP from donor 1 alone (A1-curve a, A2-curve a, B-curve a) no aggregation occurred, but in samples with added serum from donor 2 (A1-curve b, A2-curve b) or donor 3 (B-curve b), the added isolate induced platelet aggregation. This transferability of aggregation with serum was further examined through preincubation of serum with either the isolate tested in aggregation, another SBSEC isolate or an E. faecalis strain to absorb specific IgG. If serum from donor 2 was pretreated with SGG2 bacteria (A1-curve c, A2-curve c), the serum lost the ability to support aggregation of platelets from donor 1. However, if serum instead was pretreated with E. faecalis (A-curve d) or SGP1 (B-curve d), the ability to aggregate remained. With serum from donor 3, incubation with the tested isolate SII1 led to loss of aggregation (B-curve c), as did incubation with isolate SGG2 (B-curve d).

### Platelet activation by SBSEC.

**FIG 4:**
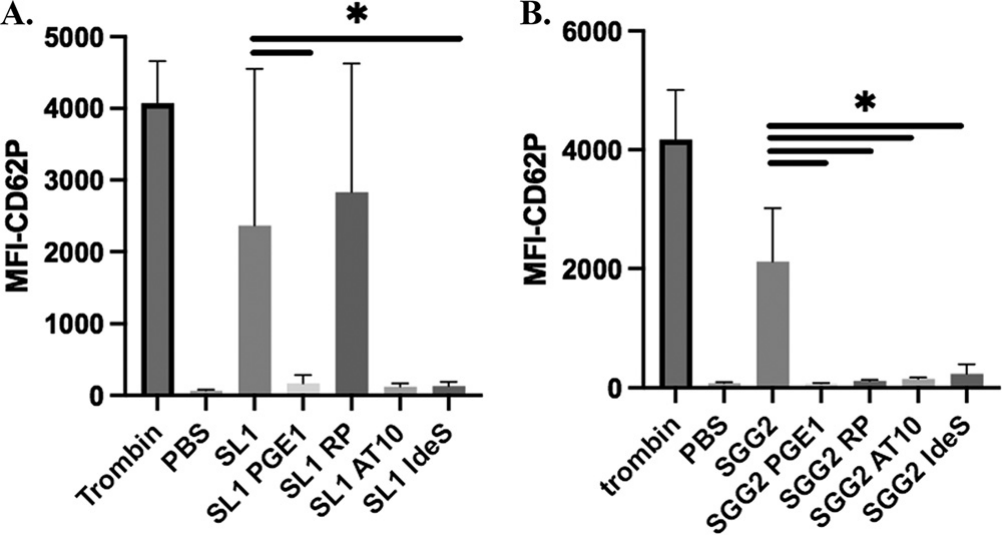
Platelet activation by SBSEC isolates. 3.3 × 10^6^ bacteria were added to PRP and upregulation of CD62P to the platelet surface, a marker of platelet activation, was studied using flow cytometry. The *y* axis represents median fluorescence intensity (MFI). Thrombin was used as positive control and buffer (PBS) as negative control. Bacteria were added to either PRP alone or to PRP pretreated with prostaglandin E_1_ (PGE_1_), ReoPro, AT10, or IdeS. The activation of platelets was studied adding SL1 to PRP from donor 1 (A, *n* = 7) and SGG2 to PRP from donor 2 (B, *n* = 3). The data presented are median values with range. Statistical analysis was made using Mann-Whitney U-test for results presented in Fig. 4A and Student's *t* test for Fig. 4B. (*) represents statistical significance (*P* ≤ 0.05).

Activation of platelets by SBSEC was determined using two-color flow cytometry following addition of either bacteria, thrombin, or buffer alone (Fig. 4). In samples with only buffer added to PRP, there was very little platelet activation (median fluorescence intensity [MFI] < 100). The positive-control thrombin generated strong platelet activation (median MFI > 3,800). As a direct comparison to aggregometry, SL1 was studied in donor 1, SGG2 in donor 2, and SII1 in donor 3. In donor 1, SL1 added to PRP resulted in platelet activation with a median MFI of 1,381 (*n* = 7), as did SGG2 in donor 2 (median MFI 2,205, *n* = 3). Though different concentrations of bacteria were tested, no platelet activation could be detected in tests with PRP from donor 3 (data not shown). The bacterial activation was also studied in PRP pretreated with blockers. In donor 2, activation by SGG2 was significantly inhibited by PGE_1_, Reopro, IdeS, and AT10, indicating a role for both IgG and the fibrinogen receptor. For SL1, platelet activation was significantly inhibited by PGE_1_ and IdeS but with addition of Reopro no inhibitory effect could be seen (median MFI = 3,337). Pretreatment with AT10 (*n* = 4) led to lower platelet activation similar to samples with IdeS and PGE_1_, although not statistically significant compared to activation in untreated samples (*P* = 0.08).

## DISCUSSION

SBSEC isolates are common pathogens in IE. Platelet activation and aggregation is a well-studied virulence mechanism of many IE pathogens, yet little is known about the interaction between SBSEC and platelets. This study suggests that platelet activation and aggregation by SBSEC is host and isolate dependent and that the IgG-platelet Fc-receptor pathway is necessary for this interaction.

From the aggregometry results of the 26 tested isolates, a variation could be seen between the donors. In donor 1, only four isolates induced aggregation, while in donor 3, 21 isolates induced aggregation. Many isolates induced aggregation in one or two donors but not all three, suggesting that aggregation is not only a matter of bacterial properties, but also dependent on host factors. This is consistent with previous findings about aggregation induced by *Sg gallolyticus* (28). To determine which host factors are of importance, a larger study with more donors would be required.

Both platelet aggregation and activation by SL1 and SGG2 were shown to be IgG dependent because IgG cleavage (IdeS) inhibited aggregation and both IdeS and Fc receptor blockade (AT10) inhibited activation. Our finding that the capacity of isolates to induce aggregation is transferable through serum also supports this claim, showing that a factor in serum was needed for the bacteria to aggregate the platelets. Furthermore, in the experiments with serum from donor 2 (Fig. 3A1 to A2), the capacity for aggregation was only diminished when serum was pretreated with SGG2 and not with the other bacteria tested, indicating that a plasma factor specific for SGG2 was required for aggregation to occur. These results suggest that aggregation by SGG2 is dependent on the presence of specific IgG toward the bacteria. For isolate SII1 and serum from donor 3 (Fig. 3B), aggregation was also transferable by serum, but the same specificity could not be shown because preincubation with both SII1 and SGG2 led to loss off aggregation. A central role of bacteria-specific or species-specific IgG in the bacteria–platelet interaction also corresponds well to the aggregometry results showing a large variation between the donors.

The isolates tested belonged to four different species or subspecies of the SBSEC (see Table 1). The three additional (sub)species in the complex are rarely involved in human infections, and therefore, are less relevant for this study (29). No clear difference could be seen between the different species regarding aggregation, with at least two isolates of each tested subspecies inducing aggregation in one or more donors. The rapid aggregation that was seen in donor 1 was induced by an isolate of *S. lutetiensis* (SL1) and an isolate of *S. infantarius* ssp*. infantarius* (SII6).

Blockade of the platelet fibrinogen receptor using ReoPro inhibited aggregation by all three isolates in their specific donor. This was expected because this receptor is necessary for bridging platelets into aggregates (30), and blockage of the receptor would inhibit aggregation even if platelets had been activated by the bacteria. However, when platelet activation was studied by flow cytometry, addition of Reopro only inhibited activation by SGG2 in donor 2, and not by SL1 in donor 1. The results indicate that a dual signaling is needed for platelet activation by SGG2. This has also been described for other IE pathogens (22, 31, 32). Some of these bacteria have also been shown to bind fibrinogen themselves (31, 32), thus enabling fibrinogen binding to the inactive GPIIb/IIIa receptor. However, when examining protein binding using the plasma-absorption experiment (described in the Materials and Methods section), we could not observe fibrinogen binding for SGG2 or SL1 (data not shown). It is possible that the binding of IgG to the Fc-receptor enables the conformational change of GPIIb/IIIa to its active state, allowing it to bind fibrinogen from plasma which leads to full platelet activation.

For SL1, the results indicate that activation of platelets in donor 1 is a process that happens solely via the Fc-receptor, or possibly via the Fc-receptor and an additional signaling pathway not studied here. This other pathway could, for example, be by activation of the complement system or a direct binding of the bacteria to the platelet. However, platelet aggregation induced via the complement system usually exhibits a longer lag-time than the very rapid aggregation induced by SL1 in donor 1 (33). For S. sanguinis, the strains binding directly to the platelet GPIb receptor induce a rapid aggregation, while isolates that use other pathways cause aggregation with a longer lag-time (19). On the other hand, the rapid aggregation induced by SL1 was only apparent in donor 1 and, if indeed a direct binding is involved, this would involve a platelet structure specific for donor 1, which appears unlikely. A larger study involving more donors would help to investigate this matter.

There are several factors in these *in vitro* experiments that differ from the environment where platelets and bacteria would interact during IE. One factor is the use of heat-killed bacteria, which obviously can affect the cell and protein structures. The decision of using heat-killed bacteria was made for reasons of practicality and reproducibility, as it then was possible to re-use the same batch of bacterial solution for several experiments. This also reduced the variation due to different growth phases of the bacteria. Aggregation by live SL1 in donor 1 was also studied using aggregometry as described above, showing that the live isolate did indeed induce rapid aggregation.

Another difference is that the interactions studied here were under static conditions, while platelets and bacteria *in vivo* interact under shear conditions. For S. aureus, the structures proven to induce platelet aggregation under static conditions have also been demonstrated to induce aggregation under high-shear conditions (34, 35), whereas for S. sanguinis the direct interaction between SrpA and the platelet GPIb receptor was only active during low-shear conditions (19). Veloso et al. (27) demonstrated that antiplatelet medication had a protective effect against *Sg gallolyticus* endocarditis in animal models, suggesting that the bacteria–platelet interaction is relevant *in vivo*. Whether this interaction involves the mechanisms described in this report remains to be determined.

In conclusion, this study reports that different subspecies of SBSEC isolates mediate aggregation and activation of human platelets in an IgG dependent manner. For one isolate, activation was also dependent on the GPIIb/IIIa receptor. A more detailed knowledge about the interactions of platelets with SBSEC and other bacteria could lead to the development of new therapeutics against diseases such as IE.

## MATERIALS AND METHODS

### Bacteria and culture conditions.

Twenty-six SBSEC blood isolates from patients with bacteremia were collected from the diagnostic laboratory of Clinical Microbiology, Region Skåne, Lund, Sweden. Subspecies were determined through whole-genome sequencing as previously described (29). S. pyogenes strain AP1 was used as a comparative control strain (22).

Bacteria were cultivated overnight in 10-mL Todd-Hewitt broth at 37°C with 5% CO_2_ and were harvested by centrifugation at 2,500 × *g* and then washed twice in phosphate-buffered saline (PBS) before being resuspended in 10 mL of the same buffer. The bacteria were heat-killed for 5 min at 80°C. Flow cytometry was applied to determine the relative concentrations based on events per microliter of sample. Heat-killed bacteria diluted 1:100 were run on a BD AccuriC6 Plus flow cytometer with volumetric counting. Events/μL were measured within the bacterial population gated by cell size (forward scatter) and granularity (side scatter). Based on these measurements, the undiluted solutions were adjusted to a concentration of 3.3 × 10^8^ bacteria/mL. Bacterial binding of plasma proteins was studied using plasma absorption and gel electrophoresis, as described in (36).

### Platelet preparation.

Blood samples were collected from three healthy donors (one female, two males; age 25 to 47) who had not taken antiplatelet medication in the previous 10 days. A 4.5-mL sample of blood was collected in tubes containing 3.2% trisodium citrate (blood to additive ratio 9:1). Centrifugation at 150 × *g* for 10 min produced an upper layer of platelet rich plasma (PRP) which was collected. Additional centrifugation at 2,000 × *g* for 10 min produced an upper layer of platelet poor plasma (PPP), which was also collected. Sampling of blood from volunteers had been approved by the local ethics committee (approval 2015/801).

### Aggregometry.

A four-channel aggregometer (Chrono-Log 490 4 + 4) was used to assess aggregation of platelets. A total of 500 μL of PPP was placed in the reference well to establish a baseline of 100% transmission. A change of light transmission of ≥ 40% was considered as aggregation. This limit was set to separate what was considered true aggregation from samples that showed a slow, limited increase in light transmission in the aggregometer during the observation time but in which no visual aggregation could be seen. To compare the different lag times for aggregation to occur, time from addition of bacteria to the point of 50% of final change in light transmission (T_50_) was measured. In the test channels, 250 μL of PRP was used. Different amounts of bacteria were added to optimize the bacteria–platelet ratio. In the final experiments,10 to 20 μL of bacteria (3.3 × 10^8^ bacteria/mL in flow cytometry) was added to the wells and the platelet reaction was monitored for a maximum of 25 min. As a positive control, 1 μL of soluble collagen I (Triolab, #385) was added to 250 μL PRP. In some experiments, the PRP was treated with either IgG-degrading enzyme (IdeS [37], final concentration 20 μg/mL), prostaglandin E_1_ (PGE_1_, Sigma, #P5515, final concentration 1 μM), or ReoPro (Centocor Biopharmaceutical, #29747B, final concentration 20 μg/mL) and incubated for 15 min at 37°C before being added to the wells. For one experiment, 25 μL of serum from a donor reacting to a certain isolate was added to 250-μL PRP from a nonreactive donor and tested for aggregation. For depletion of bacteria-specific IgG, 50 μL of serum was preincubated with 20 μL of a 2 × 10^10^ bacteria/mL solution for 20 min in 37°C. Following centrifugation at 16 800 × *g* for 2 min, 25 μL of the supernatant was added to 250 μL PRP followed by determination of aggregation. Results were analyzed using Aggro/link Opti8 software.

### Determination of platelet activation by SBSEC.

Platelet activation was studied using two-color flow cytometry. CD42- and CD62-specific antibodies were used to identify the platelet population and to detect platelet alpha granule surface expression, respectively. Then, 20 μL of PRP was added to 30 μL HEPES buffer pH 7.4. A total of 10 μL of 3.3 × 10^8^ bacteria/mL solution was added, and after 25 min, fluorochrome conjugated antibodies (10 μL CD42PerCP #340537 and 5 μL CD62PE #348107, both from BD Biosciences) were added. After additional incubation for 10 min, staining was terminated by adding 500-μL PBS, and the samples were run in a BD AccuriC6 Plus flow cytometer in logarithmic mode with gate setting for the CD42 positive platelet population. In this gate, 50,000 cells were registered and analyzed using BD CSampler Plus software. The amount of surface CD62P in the platelet population was measured and presented as MFI. As positive and negative controls thrombin (final concentration 1 U/mL, Triolab#386) or PBS were added instead of bacteria and incubated for 15 min before addition of antibodies. In some samples, the PRP was pretreated with specific blockers or enzymes—1 μM PGE_1_, 20 μg/mL Reopro, 50 μg/mL AT10 (Bio-Rad, #MCA1075), or 20 μg/mL IdeS—and incubated for 15 min at 37°C.

### Statistical analysis.

Statistical analysis of the results was made using either Mann-Whitney U-test or Student's *t* test. A *P* value ≤ 0.05 was considered statistically significant.

## Supplementary Material

Reviewer comments
